# Abordagem ao Paciente com Endocardite Infecciosa e Complicação Neurológica – O Grande Dilema que Persiste até Hoje

**DOI:** 10.36660/abc.20210198

**Published:** 2021-04-08

**Affiliations:** 1 Universidade Federal do Rio de Janeiro Hospital Universitário Clementino Fraga Filho Rio de JaneiroRJ Brasil Hospital Universitário Clementino Fraga Filho, Universidade Federal do Rio de Janeiro, Rio de Janeiro, RJ - Brasil.; 2 Universidade Estácio de Sá Faculdade de Medicina Rio de JaneiroRJ Brasil Faculdade de Medicina, Universidade Estácio de Sá, Rio de Janeiro, RJ - Brasil.; 3 Universidade Federal Fluminense Hospital Universitário Antonio Pedro NiteróiRJ Brasil Hospital Universitário Antonio Pedro, Universidade Federal Fluminense, Niterói, RJ – Brasil.

**Keywords:** Endocardite Bacteriana/complicações, Mortalidade, Complicações Neurológicas, Insuficiência Renal, Próteses Valvulares Cardíacas, Pandemia/complicações, Próteses e Implantes, Envelhecimento, Embolização

A endocardite é uma doença extremamente desafiadora em seu diagnóstico, pela diversidade de sua apresentação e também na conduta terapêutica, o que torna necessária a existência de um time de endocardite, constituído por profissionais de diversas especialidades. É de fundamental importância a presença de infectologista, microbiologista clínico, cardiologista, cirurgião cardíaco, neurologista e imagenologista e desejável a assistência de outras especialidades que dependem de cada caso. Sua incidência tem aumentado nas últimas décadas, principalmente em decorrência do envelhecimento da população, pelo crescente número de indivíduos em tratamento renal substitutivo, pela maior frequência de pacientes portadores de próteses valvares e de dispositivos elétricos cardíacos implantados e, também, devido aos avanços tecnológicos incorporados nos métodos diagnósticos e terapêuticos invasivos, além da atual “epidemia” do uso de drogas recreativas intravenosas, o que constitui, em alguns países, um grave problema de saúde pública. Apesar de todo os avanços obtidos no diagnóstico e na terapêutica da endocardite, sua mortalidade permanece assustadoramente alta. A persistência dessa grande quantidade de óbitos talvez possa, em parte, ser explicada pelo aumento do número de pacientes mais idosos, frágeis, com múltiplas comorbidades e portadores de próteses.

O artigo “Complicações neurológicas em pacientes com endocardite infecciosa: perspectivas de um centro terciário”^1^ tem como principais objetivos avaliar os preditores de complicações neurológicas em pacientes com endocardite infecciosa e os preditores de mortalidade neste grupo, e comparar os resultados do tratamento clínico com o clínico-cirúrgico, tanto na população de pacientes estudados quanto estratificar o grupo de pacientes com complicações neurológicas.

Na coorte de Alegria et al.,^1^ os fatores predisponentes independentes para o desenvolvimento de complicações neurológicas foram diabetes e ausência de febre na apresentação, dado este interessante, pois o diabetes é pouco citado como fator preditivo de embolização cerebral em pacientes com endocardite, embora tenha sido mencionado na European Society of Cardiology (ESC)^2^e constitua uma das variáveis da calculadora desenvolvida por Hubert et al.,^3^ para avaliar o risco de embolização nos pacientes com endocardite. A ausência de febre na admissão não é relacionada na literatura a complicações neurológicas, mas este achado é curioso, já que pode demonstrar um atraso no diagnóstico da endocardite e, por conseguinte, um tempo mais prolongado até o início da antibioticoterapia adequada, o que aumenta as chances de uma embolização para o sistema nervoso central (SNC), haja vista que a maior parte das complicações neurológicas ocorre antes da internação ou durante a primeira semana de antibioticoterapia. Tal complicação diminui, significativamente, após a segunda semana da terapia antimicrobiana. Outro fator preditor de embolização, citado pelos autores, foi a idade do paciente, ainda que este não tenha apresentado significância estatística na análise multivariada. É importante observar que, na maior parte das coortes, inclusive na de Alegria et al.,^1^ a idade mais avançada se relaciona com um menor risco para embolização cerebral nos pacientes com endocardite.^4^^,^^5^ No entanto, na calculadora de Hubert et al.,^3^ a idade superior a 70 anos está associada a um maior risco de embolização.

A endocardite que compromete a válvula mitral e o microrganismo causador da infecção ser *S. aureus*, fatores predisponentes para o desenvolvimento de complicações neurológicas classicamente descritos na maioria das publicações, curiosamente, não foram observados nesta coorte.^4^^,^^6^ O tamanho da vegetação, o principal fator predisponente para embolização, infelizmente não foi avaliado nesta coorte, já que a mensuração das vegetações não foi feita em todos os pacientes.

Alegria et al.^1^ abordam um dos dilemas mais angustiantes com que o time de endocardite pode se deparar: a tomada de decisão frente ao paciente que apresenta alterações neurológicas consequentes da endocardite e que persiste com vegetação de alto potencial emboligênico ou que desenvolve uma complicação potencialmente fatal, cuja cirurgia cardíaca constitui o único tratamento possível. As três principais complicações que requerem o tratamento cirúrgico são a deterioração hemodinâmica, a prevenção de embolização ou de sua recorrência e a infecção persistente. A questão não se restringe à decisão de submeter ou não o paciente a uma cirurgia, mas também quanto ao momento ideal para o procedimento.

Quando a indicação cirúrgica se dá pela deterioração hemodinâmica, mesmo com todo o risco da progressão da lesão neurológica, não resta outra opção que não seja fazê-la o quanto antes, pois o desfecho fatal, sem a intervenção cirúrgica, é bem conhecido. O mesmo ocorre quando a indicação cirúrgica é a persistência da infecção, porém, a maior preocupação surge quando a indicação do tratamento cirúrgico se dá para prevenir a recorrência de embolização para o SNC. Nesta situação, além do risco inerente do procedimento cirúrgico, que também está presente quando as indicações do procedimento são a deterioração hemodinâmica e a persistência da infecção, ainda existe o risco, potencialmente fatal, do agravamento do quadro neurológico. Não operar e correr o risco de o paciente vir a apresentar um novo quadro de embolização que pode ser fatal, ou fazer o procedimento com o risco de a lesão cerebral progredir e matar o paciente?

Ainda que já se tenha decidido pela cirurgia, outro ponto muito controverso na literatura médica é o momento em que ela deve ser realizada. Os autores relatam que a média de tempo entre o diagnóstico e a cirurgia foi de quatro semanas (36 dias). Eles comentam que este seria o intervalo necessário para evitar o agravamento da complicação neurológica causada pela heparinização necessária para a extracorpórea, o que evidencia uma postura concordante com as diretrizes internacionais atuais.

O último guideline europeu publicado recomenda adiar a cirurgia cardíaca por ao menos quatro semanas na presença de hemorragia intracraniana, a fim de evitar que, com a heparinização durante a CEC, haja um aumento da área com sangramento ou a conversão de um infarto isquêmico em hemorrágico. Além disto, o fluxo não pulsátil da CEC e a hipotensão durante a cirurgia podem prejudicar a circulação cerebral, promovendo a extensão da área de um infarto cerebral. Os autores desta diretriz consideram que os danos potenciais que a CEC pode causar sejam superiores ao benefício que a cirurgia é capaz de trazer; no entanto, essas recomendações estão baseadas na opinião de especialistas.

Várias publicações da última década referem que a presença de complicações neurológicas assintomáticas ou de ataques isquêmicos transitórios não aumentam o risco de complicações neurológicas no pós-operatório e que, portanto, a cirurgia cardíaca pode ser realizada a qualquer momento.^7^ No caso de um infarto cerebral de pequena extensão e com pouca repercussão neurológica, as recomendações quanto ao momento da cirurgia cardíaca são controversas: há quem recomende a cirurgia uma a duas semanas após o evento, há quem preconize que o intervalo poderia ser menor que 7 dias e os que consideram que a cirurgia deva ser realizada nas primeiras 72 horas e que a chance de complicações seria maior após este intervalo.^8^^,^^9^O estudo de García-Cabrera et al.,^4^ em 2013, é concordante com a revisão sistemática da literatura de Tam et al.,^10^ que concluiu que os pacientes com acidente vascular encefálico (AVE) isquêmico podem se beneficiar de um atraso de 1 a 2 semanas na cirurgia e os com evento hemorrágico, mais de 21 dias.^4^^,^^10^ No entanto, as publicações mais recentes continuam a preconizar intervalos mais curtos.^11^^,^^12^

O grande receio se dá quando há um comprometimento neurológico importante ou existe hemorragia intracraniana. Nessas situações, alguns pesquisadores recomendam não submeter o paciente ao procedimento cirúrgico ou realizar a cirurgia cardíaca após um mês, em concordância com as diretrizes internacionais atuais. Porém, muitas publicações recentes não encontraram associação entre a presença de hemorragias cerebrais ou de infarto extenso com uma chance significativamente maior de complicação neurológica no pós-operatório,^5^^,^^12^ mas, como já mencionado, há que se ter cautela na interpretação destes resultados. Mesmo que a população total de pacientes avaliados por estes autores não seja pequena, após as estratificações, o número de participantes em cada grupo a ser analisado acaba sendo muito reduzido, além da possibilidade de haver viés de seleção. Atualmente, um número expressivo de autores continua recomendando um intervalo de pelo menos 21 dias entre o evento hemorrágico e a cirurgia, a não ser que o atraso da cirurgia ponha em risco a vida do paciente.^12^

Na coorte de Alegria et al.,^1^ os pacientes que foram submetidos à cirurgia apresentaram uma taxa menor de mortalidade quando comparados aos que foram tratados exclusivamente com antimicrobianos, o que é concordante com a maior parte da literatura médica atual.^13^ Também não foi encontrada diferença na mortalidade dos pacientes com ou sem complicações neurológicas, em contraste com a maior parte dos escritos, como os próprios autores chamam a atenção.^4^^,^^5^^,^^14^

Quando analisaram os dados após a estratificação do grupo de pacientes com complicações neurológicas e submetido ao procedimento cirúrgico com o grupo que foi tratado apenas com antibioticoterapia, eles observaram uma taxa menor de mortalidade no grupo submetido ao procedimento cirúrgico, o que é concordante com a maioria das publicações mais recentes.^5^ No entanto, os autores chamam a atenção para a possibilidade de um viés de seleção.

Quanto aos fatores independentes de mortalidade nos pacientes com endocardite e complicação neurológica, os autores observaram que apenas a infecção pelo vírus HIV se mostrou estatisticamente significativa; porém, este resultado pode não se repetir em outras coortes, haja vista que apenas dois pacientes eram portadores da síndrome e apresentavam complicações neurológicas da endocardite.

Para concluir, a análise da coorte de Alegria et al.,^1^ apesar das limitações já citadas pelos autores, apresenta resultados extremamente interessantes, como apontar a diabetes como fator preditivo para a embolização e salientar a relação da infecção pelo vírus HIV, de forma independente, à mortalidade, além de apresentar, de maneira bastante detalhada, aspectos ligados ao tratamento cirúrgico em pacientes com endocardite e complicações neurológicas.

Não existem, até o momento, dados que permitam a criação de recomendações mais robustas quanto a abordagem dos pacientes com endocardite e que desenvolveram uma complicação neurológica. As diretrizes podem ajudar, mas a tomada de decisão deve ser realizada pelo time de endocardite, ponderando as características particulares de cada paciente.
