# Supplementary material for: Abordagem ao Paciente com Endocardite Infecciosa e Complicação Neurológica – O Grande Dilema que Persiste até Hoje
Source: Arq Bras Cardiol. 2021 Apr 8;116(4):692–4. [Article in Portuguese] doi: 10.36660/abc.20210198 (PMC8121385; doi:10.36660/abc.20210198)
Supplement: Translation

# Translation: Managing Patients with Infectious Endocarditis and Neurological Complication — The Big Dilemma that Persists Until these Days

**Affiliations:** 1 Rio de JaneiroRJ Brazil Hospital Universitário Clementino Fraga Filho, Universidade Federal do Rio de Janeiro, Rio de Janeiro, RJ - Brazil.; 2 Rio de JaneiroRJ Brazil Faculdade de Medicina, Universidade Estácio de Sá, Rio de Janeiro, RJ - Brazil.; 3 NiteróiRJ Brazil Hospital Universitário Antonio Pedro, Universidade Federal Fluminense, Niterói, RJ – Brazil.

**Keywords:** Endocarditis, Bacterial/complications, Mortality, Neurologic, Complications, Renal Insufficiency, Heart Valve Prosthesis, Pandemics/complications, Aging, Embolization

Endocarditis is an extremely challenging disease, both regarding diagnosis and due to the diversity of its presentation, as well as its management, which requires an endocarditis team including professionals from different specialties. An infectious disease specialist, clinical microbiologist, cardiologist, cardiac surgeon, neurologist and imaging specialist are of the essence, and assistance from other specialists is also desirable depending on each case. Its incidence has been increasing in recent decades, especially due to population aging, the growing number of individuals undergoing renal replacement therapy, the higher frequency of patients with prosthetic valves and cardiac electrical devices and also due to the technological advances in invasive diagnostic and therapeutic methods, in addition to the current “epidemic” of recreational intravenous drugs, which have become a serious public health problem in some countries. Despite all the progress made in the diagnosis and treatment of endocarditis, its mortality remains alarmingly high. The persistence of this high mortality may be partially explained by the increase in the number of older fragile patients with multiple comorbidities and patients with prostheses.

The main objectives of the article “ Neurological Complications in Patients with Infective Endocarditis: Insights from a Tertiary Centre”^1^ are to evaluate the predictors of neurological complications in patients with infective endocarditis, the predictors of mortality in this group, and to compare the results of clinical treatment with a general practitioner and a surgeon, both among the study population, and stratifying the group of patients with neurological complications.

In the Alegria et al.^1^ cohort, the independent predisposing factors for the development of neurological complications were diabetes and the absence of fever at presentation. This is an interesting information, as diabetes is little cited as a predictive factor for cerebral embolization in patients with endocarditis, although it has been mentioned in the European Society of Cardiology (ESC)^2^ and is one of the variables in the calculator developed by Hubert et al.,^3^ to assess the risk of embolization in patients with endocarditis. The absence of fever on admission is not mentioned in the literature as being related to neurological complications. However, this is an intriguing finding, as it may be reflecting delayed diagnosis of endocarditis and, therefore, a longer time stretch until initiation of proper antibiotic therapy, increasing the chances of embolization to the central nervous system (CNS), since most neurological complications occur before hospitalization or during the first week of antibiotic therapy, significantly decreased after the second week of antimicrobial therapy. Another predictor of embolization mentioned by the authors was the patient’s age, although it did not present statistical significance in the multivariate analysis. It is important to note that in most cohorts, including that of Alegria et al.,^1^ older age is related to lower risk for cerebral embolization in patients with endocarditis.^4^^,^^5^ However, in the calculator by Hubert et al.,^3^ age over 70 is considered to be associated with an increased risk of embolization.

Interestingly, endocarditis involving the mitral valve and the infecting microorganism being S. aureus, predisposing factors for the development of neurological complications, classically described in most publications, were not found in this cohort.^4^^,^^6^ The size of the vegetation, which is the main predisposing factor for embolization, was unfortunately not evaluated in this cohort, as the vegetation was not measured in all patients.

Alegria et al.^1^ address one of the most distressing dilemmas that the endocarditis team may face: decision-making for patients with neurological abnormalities resulting from endocarditis and persisting vegetation with high embolic potential or a potentially fatal complication, whose cardiac surgery is the only possible treatment. The three main complications that require surgical treatment are hemodynamic deterioration, prevention of embolization or its recurrence, and persistent infection. The issue is not restricted to the decision to submit the patient to surgery or not, but also as to the ideal moment for the intervention.

When surgical indication is due to hemodynamic deterioration, even with all the risk of progression of neurological injury resulting from cardiopulmonary bypass (CPB), because a fatal outcome, without surgical intervention, is well known. The same occurs when surgical indication is persistence of infection. However, the greatest distress arises when indication for surgical treatment is to prevent the recurrence of embolization to the CNS, as, in this situation, in addition to the inherent risk of the surgical procedure (which is also present when the indications for the procedure are hemodynamic deterioration and persistence of infection), there is still a potentially fatal risk of worsening the neurological condition. In this situation, even if surgery has been chosen, another very controversial point in the literature is the moment when it should be performed.

The authors report that the average time between diagnosis and surgery was 4 weeks (36 days) and that this would be the time interval required to avoid worsening the neurological complication as a result of the necessary heparinization for cardiopulmonary bypass, which is consistent with the current international guidelines.

The latest European guideline recommends postponing cardiac surgery for at least 4 weeks in the presence of intracranial hemorrhage to prevent heparinization during cardiopulmonary bypass from increasing bleeding area or to prevent the conversion of ischemic infarction to hemorrhagic infarction. Besides, the non-pulsatile flow of cardiopulmonary bypass and hypotension during surgery may impair cerebral circulation and promote extension of cerebral infarction area. The authors of the guideline consider that the potential damage that cardiopulmonary bypass may cause is greater than the benefit that surgery can bring, but these recommendations are based on expert opinion.

Over the last decade, several publications reported that the presence of asymptomatic neurological complications or transient ischemic attacks does not increase the risk of neurological complications in the postoperative period, and that, therefore, cardiac surgery can be performed at any time.^7^ When the patient has a small cerebral infarction with little neurological repercussion, they recommend that cardiac surgery be performed after 1 to 2 weeks after the neurological event, and other articles recommended that the interval could be less than 7 days. Others considered that surgery should be performed within the first 72 hours after the onset of the neurological condition and that after this interval the possibility of complications would be greater.^8^^,^^9^ However, the study by García-Cabrera et al.,^4^ in 2013, is consistent with the systematic literature review carried out by Tam et al.,10 where they concluded that patients with ischemic stroke can benefit from a delay of 1 to 2 weeks for surgery and those with a hemorrhagic event, more than 21 days.^4^^,^^10^ However, the most recent publications continue to recommend shorter intervals.^11^^,^^12^

The greatest fears are severe neurological impairment or intracranial hemorrhage. In these situations, some researchers recommend not to operate the patient or to perform cardiac surgery after 1 month, in line with the current international guidelines. However, many recent publications have not found an association between the presence of cerebral hemorrhage or extensive infarction with significantly greater chances of neurological complications in the postoperative period,^5^^,^^12^ but, as mentioned before, care should be taken in the interpretation of these results, because, even if the total population of patients evaluated by these authors is not small, after stratifications, the number of participants in each group to be analyzed ends up being too small, in addition to the possibility of selection bias. Presently, a significant number of authors continue to recommend an interval of at least 21 days between the hemorrhagic event and the surgery, unless a surgery delay puts the patient’s life at risk.^12^

In the cohort of Alegria et al.,^1^ operated patients had lower mortality compared to those treated exclusively with antimicrobials, which is in line with most of the current literature.^13^ Alegria et al.^1^ found no difference in the mortality of patients with or without neurological complications, which was different from most of the literature, as the authors themselves argued.^4^^,^^5^^,^^14^

When they compared the data after stratification of the group of patients with neurological complications who underwent the surgical procedure to the group of patients treated with antibiotic therapy only, they found lower mortality in the group undergoing the surgical procedure, which is in line with most of the latest publications,^5^ but the authors call attention to the possibility of a selection bias.

Regarding the independent factors of mortality in patients with endocarditis with neurological complications, the authors found that only HIV infection was shown to be statistically significant, but this result may not be repeated in other cohorts, since only two patients had HIV infection and neurological complications from endocarditis.

In conclusion, despite the limitations mentioned by the authors, the cohort of Alegria et al.^1^ presents extremely interesting results, such as diabetes as a predictive factor for embolization and HIV infection as independently related to mortality. It also presents, in a very detailed way, aspects related to surgical treatment in patients with endocarditis and neurological complications.

Finally, there are, to date, no data that allow the creation of more robust recommendations regarding the approach of patients with endocarditis who have developed a neurological complication. The guidelines can help, but the decision must be made by the endocarditis team considering the particular characteristics of each patient and each case.
